# Prevalence and correlates of alcohol use in a central Nepal district: secondary analysis of a population-based cross-sectional study

**DOI:** 10.1017/gmh.2018.28

**Published:** 2018-11-13

**Authors:** S. D. Rathod, N. P. Luitel, M. J. D. Jordans

**Affiliations:** 1 Department of Population Health, London School of Hygiene and Tropical Medicine, London, UK; 2 Research Department, Transcultural Psychosocial Organization Nepal, Kathmandu, Nepal; 3 Centre for Global Mental Health, Institute of Psychiatry, Psychology and Neuroscience, King's College London, London, UK; 4 Research and Development Department, HealthNet TPO Amsterdam, Amsterdam, The Netherlands

**Keywords:** Adult, alcohol drinking, Nepal, primary health care, stigma

## Abstract

**Background.:**

As reported from studies conducted in Nepal, between 15% and 57% of adults had ever consumed alcohol and between 1.5% and 25% of adults have alcohol use disorders (AUD). Few studies in Nepal have identified the correlates of consumption or described the help-seeking patterns and stigma among those affected with AUD.

**Methods.:**

Interviewers administered the Alcohol Use Disorders Identification Test (AUDIT) as part of population-based surveys of adults in Chitwan District between 2013 and 2017. We conducted a secondary analysis to identify sociodemographic and health-related correlates of recent alcohol consumption using the χ^2^ test, to identify correlates of total AUDIT scores among men who drink using negative binomial regression, and to describe the treatment-seeking and stigma beliefs of men with AUD.

**Results.:**

Over half (53.7%, 95% CI 50.4–57.0) of men (*n*  =  1130) recently consumed alcohol, and there were associations between being a drinker with age, religion, caste, education, occupation and tobacco use. Nearly one in four (23.8%, 95% CI 20.2–27.8%) male drinkers screened positive for AUD, and AUDIT scores were associated with age, caste, marital status, occupation, tobacco use, depression, functional status and suicidal ideation. Few (13.3%, 95% CI 11.7–15.0) women (*n*  =  2352) recently consumed alcohol, and 5.3% (95% CI 3.0–9.1) of female drinkers screened positive for AUD. Among AUDIT-positive men, 38% spoke to another person about their problems and 80% had internalized stigma.

**Conclusions.:**

This study revealed that nearly one in four men who drink likely have AUD. Higher AUDIT scores were associated with depression, suicidality, dysfunctionality and internalized stigma.

## Background

Alcohol is responsible for over 5% of global burden of disease and injury (World Health Organization, 2014). Across Nepal, alcohol use varies widely: various studies have estimated that between 15% and 57% of adults had ever consumed alcohol (Dhital *et al*. 2001; Chataut *et al*. 2011; Lee *et al*. 2011; Manandhar *et al*. 2012; Luitel *et al*. 2013; Aryal *et al*. 2014; World Health Organization, 2014) and between 1.5% and 25% of adults have behaviours consistent with alcohol use disorders (AUD) (Jhingan *et al*. 2003; World Health Organization, 2014; Aryal *et al*. 2015). Similar to other low-income countries, Nepal, prior to 2014, lacked a national strategy to reduce harmful alcohol use (Riley & Cowan, 2014; World Health Organization, 2014). Yet, emerging evidence (Benegal *et al*. 2009; Nadkarni *et al*. 2017) demonstrates that it is effective and cost-effective to deliver treatment such as Brief Intervention (Babor & Higgins-Biddle, 2001) for harmful alcohol use at the primary care level in low- and middle-income countries.

Epidemiologic studies conducted in Nepal have characterized the burden and consequences of alcohol consumption in the community (Katz *et al*. 2003; Tausig *et al*. 2004; Ho-Yen *et al*. 2007; Kulkarni *et al*. 2010; Chataut *et al*. 2011; Lee *et al*. 2011; Oshiro *et al*. 2011; Manandhar *et al*. 2012; Bam *et al*. 2013; Khan *et al*. 2013; Adhikari *et al*. 2014; Vaidya & Krettek, 2014; Atteraya *et al*. 2015), and clinical studies have characterized the consumption histories of hospitalized patients (Shyangwa *et al*. 2009; Subba *et al*. 2010; Bam *et al*. 2011; Maskey *et al*. 2011*a*, *b*; Pradhan *et al*. 2012; Gyawali *et al*. 2013), but few population-based studies in Nepal have identified the correlates of alcohol consumption in the population, or have described the help-seeking patterns and barriers among those whose drinking patterns are consistent with AUD. Understanding alcohol consumption patterns will inform community engagement efforts and case detection, and with treatment provision in the public sector.

In this secondary analysis, we have described the alcohol-related features of adults in Chitwan District, Nepal, specifically relating to recent alcohol consumption and severity of alcohol use problems, and the internalized stigma beliefs of men with AUD.

## Methods

### Setting

Nepal is classified as a low-income state (Cosic *et al*. 2017), with a human development rank of 144 and life expectancy of 70 years (United Nations Development Programme, 2016). Chitwan District is in central Nepal near the India border. A relatively affluent district, the literacy rate is 77% (Central Bureau of Statistics, 2014), compared to the national average of 57% (UNDP, 2015). As of 2014, no evidence-based services for hazardous or harmful alcohol use were available in the primary health care sector Chitwan. The nearest detoxification and rehabilitation services for people who were dependent on alcohol were available in the district headquarters.

The Programme to Improve Mental Health Care (PRIME) consortium aims to improve the accessibility of treatment by integrating mental health services into the primary health care sector in low- and middle-income country settings (Lund *et al*. 2012). In consultation with Nepal Ministry of Health officials, 10 Village Development Committees (VDC) in Chitwan District, with a total population of 108 369 (Central Bureau of Statistics, 2014) were selected for the initial stage of the PRIME implementation (Jordans *et al*. 2016).

### Study design and sampling

Details of the PRIME Community Study, including the sampling plan, recruitment and questionnaire design were previously reported (Rathod *et al*. 2016). The primary aim of the Community Study was to estimate a change in population-level treatment coverage for depression and for AUD in Chitwan District, coinciding with the implementation of a district-level mental health care plan (Jordans *et al*. 2016). This secondary analysis of the Community Survey dataset – described in brief below – specifically concerned the correlates of alcohol use. Two survey rounds were completed (*n*  =  1983 in May–August 2013 and *n*  =  1499 in December 2016–March 2017), which was set to have 80% power to detect an increase in coverage from 5% to 25%. At each survey round, fieldworkers used sampling frames consisting of households enumerated by the VDC authorities and used a computer programme to select households randomly. The fieldworkers approached a member of selected households, took an inventory of adults (inventory participation rate 100%), wrote the adults’ names on individual pieces of paper, mixed up the pieces and asked a family member to pick one piece as a means of selecting the adult for recruitment into the study (consent rate 99%). An adult was eligible to participate if they were resident of the household and age 18 years or more, and ineligible if they were intellectually incapable of providing informed consent. For this secondary analysis, all participants were included, and unless otherwise indicated the data from both survey rounds were pooled. There were no missing data.

### Data collection

With participating adults, the fieldworker orally administered a Nepali-language-structured interview using a questionnaire application programmed on an Android tablet. All participants completed the questionnaire sections about basic sociodemographic characteristics, tobacco use, AUD screening, and depression screening and suicidality. Participants who were identified as AUD cases or depression cases completed disorder-specific sections about who they spoke to about the problems, and about their internalized stigma beliefs. To facilitate secondary analyses such as for this report while reducing data collection burden, selected participants completed an extended version of the questionnaire, which included sections on sociodemographic characteristics, household socioeconomic status, hospitalization and functional status. These sections were completed by all screen-positive participants and by 10% of screen-negative participants who were selected through a random number generator in the questionnaire application.

### Study measures

The tobacco and alcohol use section contained questions about current tobacco use, lifetime alcohol use and the 10-item Alcohol Use Disorders Identification Test (AUDIT) (Babor *et al*. 2001). Prior to any questions about alcohol, the questionnaire application included a script for the fieldworker to read to women and Brahmin/Chhetri caste members, acknowledging that traditionally members of these groups did not consume alcohol but that habits have changed. The AUDIT has been widely used to screen individuals for AUD for research purposes, and has been validated in Nepal (Pradhan *et al*. 2012). From the total AUDIT score, we considered a person with a score of 0 to be abstinent, ≥1 to be a recent (within 12 months) consumer of alcohol; and, using World Health Organization guidelines (Babor *et al*. 2001), we considered a score of ≥8 to be a positive screen for AUD, inclusive of the following sub-categories: 8–15 to be indicative of hazardous drinking patterns, 16–19 of harmful drinking and ≥20 of dependent drinking.

To screen for depression, we used the total score of the Patient Health Questionnaire (PHQ9) screening tool (Kroenke *et al*. 2001), which has been validated in Nepal (Kohrt *et al*. 2016). A score of 10 or more was a positive screen. For suicidal ideation, we adapted a question from the Composite International Diagnostic Interview (Robins *et al*. 1988), and question about hospitalization in the past 12 months was drawn from the Client Service Inventory Receipt (Beecham & Knapp, 2001).

PHQ9 and AUDIT screen-positive participants were asked 11 questions from the Internalized Stigma of Mental Illness (Boyd Ritsher *et al*. 2003) measure, and those who had responses of ‘agree’ or ‘strongly agree’ were considered to have that internalized stigma belief. To assess household economic status, we asked questions drawn from the Nepal Demographic and Health Survey [Ministry of Health & Population (MOHP) [Nepal] *et al*. 2012] about drinking water source, toilet access, housing amenities and household assets. We entered the dummy coded economic variables into principle components analysis using the method described by Vyas & Kumaranayake (2006) and created three equally proportioned groups from the sum of first factor weights.

We created tertiles from the World Health Organization Disability Assessment Schedule (Üstün *et al*. 2010) total score to create categories of functional status. For the question about religion, we created categories of Hindu, Buddhist and other. For caste, we combined Magar, Gurung, Newar and Tamang into a single ‘Janajati’ group, as these groups share important cultural characteristics. For marital status, we combined those who were separated, divorced or widowed into a single group of people who were post-marital. For occupation, we combined service and business workers into a group, and all other non-agricultural occupations into a group.

### Ethics

Fieldworkers gave adults written and oral information about the study and adults were asked if they had questions about the study before deciding to participate. Literate adults provided written confirmation of consent, while – due to cultural consideration against use of fingerprinting – illiterate adults provided verbal confirmation of consent. Participants who provided affirmative responses to questions about suicidality were referred to psychosocial counsellors who were mobilized as part of PRIME. The study protocol and consent procedures were approved by institutional review committees of the Nepal Health Research Council (Kathmandu, Nepal), World Health Organization (Geneva, Switzerland) and the University of Cape Town (South Africa).

### Statistical analysis

As a secondary analysis, the choice of characteristics for this analysis was guided by availability from the study questionnaire. First, we summarized the sociodemographic (i.e. age, occupation, caste, religion, marital status, household economic status) and health-related (i.e. tobacco use, depression score, functional status, suicidal ideation, hospitalization) characteristics separately for men and women in the study. Within each category, we reported the proportion of participants who had recently consumed alcohol and tested for associations using the Pearson's χ^2^ test. We noted when χ^2^ *p* < 0.05. Second, separately for men and women, we reported the proportion who had AUD (AUDIT score ≥8) and who were dependent on alcohol (AUDIT score ≥20). For the rest of the analysis, we limited the sample to men: though many women in Nepal consume alcohol (Dhital *et al*. 2001), the frequency and intensity of their consumption is substantially lower than for men (Riley & Cowan, 2014; Aryal *et al*. 2015), which was observed for this sample in a previous report (Rathod *et al*. 2016).

Third, among men who recently consumed alcohol (AUDIT ≥1), we assessed whether any sociodemographic or health-related characteristics were associated with AUDIT scores. As the dependent variable (AUDIT score) distribution was right skewed, we could not use linear regression, and instead, we used negative binomial regression (Elhai *et al*. 2008) to estimate the mean AUDIT score and 95% confidence interval for each category of the characteristic. We used a separate regression model for each characteristic of interest. We noted when the regression model's *F* test had *p* < 0.05, indicating evidence of an association for the characteristic with the AUDIT score. Finally, we tabulated the proportion of men with AUD who ‘agree’ or ‘strongly agree’ with 11 internalized stigma beliefs.

We conducted the analysis in Stata 14.1 (College Station, TX, USA). An additional text file contains the Stata code for this analysis (see Additional file 1). Aside counts, we adjusted all figures reported here for the population-based sampling design and sampling probability weights.

## Results

### Characteristics of the sample and of recent drinkers

The sociodemographic characteristics of men and women in the sample along with the proportions who recently (within past 12 months) consumed alcohol within each sociodemographic category are presented in Table 1.

**Table 1.** Sociodemographic and health-related characteristics of men and women, and proportion with recent alcohol consumption, Chitwan District, Nepal, 2013–2017

	Men			Women		
	*n* in sample (%)	Of whom drink alcohol (%)	*p* Value	*n* in sample (%)	Of whom drink alcohol (%)	*p* Value
Total	1130 (100)	53.7		2352 (100)	13.3	
*Socioeconomic characteristics*						
Age (years)						
18–29	254 (25.0)	52.4	0.001	649 (31.9)	6.8	0.001
30–39	195 (17.8)	65.2		758 (25.4)	12.1	
40–49	228 (17.5)	61.7		453 (20.5)	16.9	
50–59	206 (18.2)	63.1		281 (13.3)	21.9	
60–88	247 (21.5)	31.4		211 (8.9)	18.4	
Religion						
Hindu	927 (82.2)	51.1	0.001	1916 (80.9)	11.2	0.001
Buddhist	185 (16.3)	68.4		377 (16.5)	24.6	
Other	18 (1.5)	35.9		59 (2.6)	6.4	
Caste						
Brahmin or Chhetri	577 (51.6)	35.7	0.001	1142 (48.9)	2.3	0.001
Janajati	300 (26.4)	68.0		629 (26.4)	24.8	
Dalit	159 (13.2)	80.0		378 (15.0)	19.7	
Other	94 (8.8)	77.2		203 (9.8)	26.9	
Marital status						
Never married	184 (17.4)	49.2	0.093	166 (9.2)	4.9	0.001
Currently married	899 (79.0)	55.4		1999 (83.9)	13.5	
Separated, divorced or widowed	47 (3.7)	39.9		187 (6.9)	21.5	
Education						
Less than primary	509 (43.5)	54.3	0.001	1302 (53.7)	18.5	0.001
Completed primary	470 (42.1)	58.1		870 (37.0)	7.9	
Completed secondary	151 (14.4)	39.1		180 (9.3)	4.6	
Occupation						
Agriculture	576 (50.1)	48.6	0.008	1598 (65.4)	14.2	0.142
Service or business	253 (22.1)	57.5		247 (10.2)	9.5	
Other	301 (27.8)	60.0		507 (24.4)	12.2	
Economic group^a^						
Lower	150 (30.3)	52.4	0.830	195 (33.4)	19.3	0.033
Middle	91 (37.3)	45.3		159 (42.0)	10.2	
Higher	63 (32.3)	48.8		107 (24.6)	4.6	
*Health-related characteristics*						
Tobacco user						
No	524 (48.1)	41.7	0.001	1996 (86.1)	9.1	0.001
Yes	606 (51.9)	64.9		356 (13.9)	39.0	
Depression screening						
Negative	1085 (96.4)	53.9	0.499	2242 (95.6)	12.5	0.001
Positive	45 (3.6)	48.1		110 (4.4)	30.4	
Functioning^a^						
Higher	81 (36.5)	53.7	0.449	98 (31.6)	6.6	0.343
Average	77 (30.7)	39.4		101 (27.2)	11.7	
Lower	146 (32.9)	51.6		262 (41.2)	16.0	
Suicidal ideation						
No	1099 (97.5)	53.5	0.356	2232 (94.9)	12.7	0.003
Yes	31 (2.5)	62.8		120 (5.1)	23.5	
Use of inpatient services^a^						
No	282 (92.1)	48.8	0.885	414 (92.9)	11.5	0.627
Yes	22 (7.9)	46.0		47 (7.1)	16.5	

Counts are reported as observed in the sample, while percentage figures and *p* values were estimated with design-adjusted Pearson χ^2^ test.

There were no missing data.

a Assessed among participants who screened positive for depression or screened positive for alcohol use disorder, and a randomly selected 10% sub-sample of participants who screened negative for both.

Within the total sample of 3482, 36% were men and 64% were women. Among men, 53.7% (95% CI 50.4–57.0) had recently consumed alcohol. A quarter (25.0%) of men were aged between 18 and 29 years, and of these men, 52.4% recently consumed alcohol. Another 17.8% were aged 30–39 years, and of these, 65.2% recently consumed alcohol. Over half (51.6%) of men were in the Brahmin or Chhetri caste group, and 35.7% of these men recently consumed alcohol. Drinking was more likely among men from Janajati (68.0%), Dalit (80.0%) or other (77.2%) castes. There was evidence of association for drinking alcohol by category of age, religion, caste, education, occupation and tobacco use (all *p* < 0.05). Among women, 13.3% (95% CI 11.7–15.0) had recently consumed alcohol. Nearly a third (31.9%) of women were aged between 18 and 29 years, and 6.8% of these women recently consumed alcohol. Another 13.3% of women were aged between 50 and 59 years, and 21.9% of these women recently consumed alcohol. Almost half (48.9%) of the women were in the Brahmin or Chhetri caste, and 2.3% of these women recently consumed alcohol. Of the remaining caste groups, between 19.7% and 26.9% of women recently consumed alcohol. There was evidence of association for drinking alcohol by category of age, religion, caste, marital status, education, economic group, tobacco use, depression severity and suicidal ideation (all *p* < 0.05).

Of the men who recently consumed alcohol, nearly one in four (23.8%, 95% CI 20.2–27.8%) had AUD (AUDIT score ≥8), which is inclusive of 15.3% who had hazardous drinking, 3.1% with harmful drinking and 5.4% with dependent drinking. Of the women who recently consumed alcohol, 5.3% (95% CI 3.0–9.1) had AUDIT scores consistent with AUD, inclusive of 4.2% with hazardous drinking, 0.3% with harmful drinking and 0.3% with dependent drinking.

### Correlates of the AUDIT score

The mean AUDIT scores of men who recently consumed alcohol are presented in Table 2, and the scores are stratified by various sociodemographic- and health-related characteristics.

**Table 2.** Sociodemographic and health-related correlates of AUDIT score among men who drink alcohol in Chitwan District, Nepal, 2013–2017

	*n*	Mean AUDIT score	95% CI	*p* Value
All men who drink	618	5.5	4.9–6.1	
*Sociodemographic characteristics*				
Age (years)				
18–29	130	3.5	2.9–4.2	0.001
30–39	218	5.6	4.4–6.9	
40–49	146	6.9	5.5–8.4	
50–59	133	6.2	5.0–7.4	
60–88	81	6.0	4.6–7.7	
Religion				
Hindu	485	5.6	5.0–6.3	0.001
Buddhist	126	5.0	3.9–6.2	
Other	7	5.1	2.2–8.9	
Caste				
Brahmin or Chhetri	217	4.5	3.7–5.3	0.001
Janajati	204	5.3	4.4–6.2	
Dalit	125	6.1	4.9–7.4	
Other	72	8.2	6.3–10.5	
Marital status				
Never married	91	3.1	2.4–4.0	0.001
Currently married	504	5.9	5.3–6.6	
Separated, divorced or widowed	23	9.0	5.0–13.6	
Education				
Less than primary	282	7.0	6.1–8.1	0.001
Completed primary	274	4.7	4.0–5.4	
Completed secondary	62	3.4	2.3–4.8	
Occupation				
Agriculture	285	6.2	5.4–7.2	0.001
Service or business	149	4.4	3.6–5.3	
Non-agricultural	184	5.3	4.4–6.3	
Household economic status^a^				
Lower	118	9.0	6.6–11.7	0.001
Middle	55	5.0	3.1–7.5	
Higher	42	4.4	2.6–6.9	
*Health-related characteristics*
Tobacco user				
No	214	3.3	2.7–4.0	0.001
Yes	404	7.2	6.5–8.0	
Depression screening				
Negative	593	5.3	4.7–5.9	0.001
Positive	25	16.9	12.6–20.9	
Functioning^a^				
Higher	61	4.2	2.7–6.5	0.001
Average	56	5.7	4.3–7.7	
Lower	98	8.7	6.0–11.7	
Suicidal ideation				
No	598	5.3	4.8–5.8	0.001
Yes	20	13.8	8.7–19.4	
Use of inpatient services^a^				
No	204	6.1	4.1–7.9	0.001
Yes	11	4.9	3.0–15.9	

Counts reported as observed, while mean AUDIT scores, 95% CIs and *p* values were estimated with design-adjusted negative binomial regression.

There were no missing data.

a Assessed among participants who screened positive for depression or screened positive for alcohol use disorder, and a randomly selected 10% sub-sample of participants who screened negative for both.

Overall, the mean AUDIT score for men who consumed alcohol was 5.5 (95% CI 4.9–6.1). There was evidence of an association between age group and AUDIT scores, ranging from a mean AUDIT score of 3.5 for men aged 18–29 to a mean AUDIT score of 6.9 for men aged 40–49. AUDIT scores were inversely associated with educational attainment, with a mean score of 7.0 for men with less than primary education to 3.4 for men who completed secondary education. A similar association was evident for economic group. AUDIT scores were especially high among men who were tobacco users (mean 7.2), who screened positive for depression (mean 16.9), had lower functional status (mean 8.7) or had suicidal ideation in the past 12 months (mean 13.8).

### Talking about problems and internalized stigma

Among AUDIT-positive men, only 38% spoke to at least one other person about problems with alcohol. Of the men who spoke to another person, they were most likely to speak to a spouse/partner (64%), friend or neighbour (60%), or another family member (30%).

The internalized stigma beliefs of men with AUD are presented in Table 3.

**Table 3.** Internalized stigma beliefs endorsed among men who screen positive for alcohol use disorders (AUDIT ⩾8) in Chitwan District, Nepal, 2013–2017

‘Because of these problems with alcohol…’	*n*	‘Agree’ or ‘strongly agree’ (%)
I am disappointed in myself	71	45.6
These problems have spoiled my life	71	42.7
Others think that I can't achieve much in life	64	40.1
People discriminate against me	56	33.6
I am embarrassed or ashamed	54	33.8
People ignore me or take me less seriously	51	30.4
Nobody would be interested in getting closer to me	50	28.2
I feel out of place in the world	44	29.0
I can't contribute anything to society	39	24.9
People often patronize me, or treat me like a child	34	20.9
I need others to make most decisions for me	32	21.5

Per cent figures sum to more than 100% because participants could agree with more than one statement.

There were no missing data.

Counts are reported as observed, while percentage figures are design-adjusted.

Nearly half (45.6%) of the men with AUD say they would either ‘agree’ or ‘strongly agree’ that ‘I am disappointed in myself’. Men with AUD were also likely to agree that ‘These problems have spoiled my life’ (42.7%) or that ‘Others think I can achieve much in life’ (40.1%). Nearly one in five men with AUD (19.4%) did not agree with any of the statements.

## Discussion

Consumption of alcohol was largely a male habit in Chitwan District; among women, its use was concentrated among specific subgroups. Among men who drink, AUDIT scores were positively associated with suicidality, depression and dysfunction, and among men with AUD, feelings of internalized stigma were common. Much of the data concerning alcohol use in Nepal were generated from studies which investigated the burden and consequences of alcohol use (Katz *et al*. 2003; Tausig *et al*. 2004; Ho-Yen *et al*. 2007; Kulkarni *et al*. 2010; Chataut *et al*. 2011; Lee *et al*. 2011; Oshiro *et al*. 2011; Manandhar *et al*. 2012; Bam *et al*. 2013; Khan *et al*. 2013; Adhikari *et al*. 2014; Vaidya & Krettek, 2014; Atteraya *et al*. 2015), or were based on AUD patients in tertiary care facilities (Shyangwa *et al*. 2009; Subba *et al*. 2010; Bam *et al*. 2011; Maskey *et al*. 2011*a*, *b*; Pradhan *et al*. 2012; Gyawali *et al*. 2013); these data offer a more complete picture of the population-level epidemiologic features of alcohol use in Nepal.

The prevalence of recent alcohol consumption in Chitwan District provides further evidence of the wide variation of drinking prevalence across Nepal. Findings from where community-based studies in Nepal are that between 18% and 71% of men (Dhital *et al*. 2001; Oshiro *et al*. 2011; Luitel *et al*. 2013; Vaidya & Krettek, 2014; Atteraya *et al*. 2015) and 6.8–28% of women (Dhital *et al*. 2001; Katz *et al*. 2003; Kulkarni *et al*. 2010; Khan *et al*. 2013; Luitel *et al*. 2013; Vaidya & Krettek, 2014) recently drank alcohol. As observed here, a majority (54%) of all men drank alcohol in the past 12 months, and the behaviour was widespread across sub-groups: men between 60 and 88 years of age were least likely (31%) to drink and Dalit caste members were most likely (80%).

Among women, there were notable variations within subgroups: though 13% of all women in Chitwan were recent drinkers, the probability of drinking was substantially higher for women who used tobacco, screened positive for depression or had suicidal ideation (all >23%). These associations are suggestive of alcohol use as self-medication for mental distress, a hypothesis which can be explored with qualitative methods. Though it is not possible to establish the causal direction between alcohol use and factors such as education and economic status, the associations observed here highlight how the role of alcohol must be considered as part of any discussion of social development. Further research can establish whether drinking behaviour becomes more common among all women or within specific subgroups, and thus inform alcohol education programmes.

We found evidence that AUDIT scores were inversely associated with wellness among male drinkers. Higher AUDIT scores were evident among male drinkers who used tobacco, experienced suicidal ideation and had higher levels of dysfunction and depression. Other studies from Nepal have found alcohol use to be positively associated with sexual risk taking (Poudel *et al*. 2004; Bam *et al*. 2013), hypertension (Chataut *et al*. 2011; Khan *et al*. 2013), psychological disorders (Tausig *et al*. 2004), physical inactivity (Vaidya & Krettek, 2014), tuberculosis infection (Gyawali *et al*. 2013) and betel-quid dependency (Lee *et al*. 2012). In this district of Nepal, while only 5% of men with AUD sought treatment specifically for problems with alcohol (Rathod *et al*. 2016), it is likely that a substantial proportion of men with AUD accessed health care for these other health problems. As has recently been demonstrated in India, general service providers can offer effective counselling (Nadkarni *et al*. 2017). By addressing the alcohol problem first, the service provider will improve the prognosis for the co-morbid condition. For men with AUD who are not accessing health care, a more pro-active approach such as community-informed referral may be appropriate, and has been recently demonstrated as feasible and effective in Chitwan (Jordans *et al*. 2015).

Also notable was the pattern of alcohol consumption by age, education and household economic status. Over 60% of men aged 30–59 years consumed alcohol (*v*. 52% for 18–29 years and 31% for 60–88 years), and the AUDIT score for drinkers peaks at a mean of 7.0 for those aged 40–49 years and slightly declines thereafter. Further research can investigate the life course factors (e.g. marriage, becoming head of household) which lead men to drink more heavily in middle age. For education, men with highest level of education were least likely to drink, and those who did drink had lower AUDIT scores. Though alcohol consumption was consistent by economic status, there was an inverse association with AUDIT scores. The inverse association has been observed consistently in high-income countries, with fewer data available in low- and middle-income settings (Blas *et al*. 2010); available evidence underpinning conceptual models highlight how low socioeconomic status is both a risk factor and a consequence of alcohol consumption (Blas *et al*. 2010; World Health Organization, 2014).

Globally, health service utilization for mental health disorders varies by area and by the specific disorder; utilization is least likely for adults in low-income countries and for alcohol problems (Kohn *et al*. 2004). This pattern was confirmed in Chitwan, where only 5% of AUDIT-positive adults sought treatment for their problems (Rathod *et al*. 2016). Accordingly, clinic-based health promotion programmes are likely to have limited effect on alcohol treatment coverage in a low-income setting such as Nepal. In Chitwan, 38% of men with AUD discussed their alcohol problems with another person, and often to a friend or neighbour, and as such there is potential to promote treatment seeking through community-informed referral (Jordans *et al*. 2015). For the 62% of men with AUD who do not discuss their problems with others, further exploration of the internalized stigma beliefs is warranted. Most men with AUD (81%) endorsed at least one internalized stigma belief. It is possible that community sensitization programmes can create awareness that alcohol problems are not indicative of a personal failing, that alcohol habits are modifiable, and that discussion with a health care provider can help. Developing effective community sensitization programmes remains a key research goal for addressing the treatment gap.

This study was unique in Nepal in that it was a large community-based survey of men and women conducted over a large geographic area, with a population-based sampling design and analysis. We used a widely validated screening tool to measure alcohol use, which facilitates comparison across settings. However, this study also has important limitations. As a secondary analysis, the choice of characteristics for this analysis was guided by availability from the study questionnaire, and there may be other important risk groups for alcohol use which were not investigated here. Like many areas of Nepal, Chitwan District has a high level of seasonal labour migration (Bohra & Massey, 2009; Central Bureau of Statistics, 2014), and so we are likely to have undersampled men in this study. Male migrants are a notable risk group with regard to alcohol (Poudel *et al*. 2004; Bam *et al*. 2013) and who were less likely to be captured in this study. Though our interviewers had a special script to sensitize participants who belonged to traditionally abstinent groups (i.e. women and members of the Brahmin caste), we must also note the high levels of internalized stigma observed among those with AUD likely prevented other drinkers from acknowledging their drinking, leading to an underestimate of the prevalence of alcohol consumption. Finally, as a cross-sectional study measuring the prevalence of drinking behaviours across the population, the correlations found here are not intended to be interpreted as causal factors but as factors to inform health service provision and health promotion planning. Future research using longitudinal designs can identify the risk factors for developing AUD and can inform the development of prevention strategies.

A priority-setting exercise with expert stakeholders in Nepal came to a consensus that the burden, severity and treatability of AUD made it a strong candidate for provision of treatment services (Jordans *et al*. 2013). This study provides support for the assumptions about burden and severity, in that a large proportion of men who drink have AUD, and there is a confluence of depression, suicidality and dysfunctionality among men who drink more heavily. As AUD treatment services have now been developed (Jordans *et al*. 2016) and integrated into the primary health care sector in Chitwan District, subsequent work must focus on promoting help seeking for men with AUD, and to monitor trends in alcohol consumption over time.

## Supplementary material

For supplementary material accompanying this paper visit http://dx.doi.org/10.1017/gmh.2018.28. click here to view supplementary material
