# Supplementary material for: Prevalence and correlates of alcohol use in a central Nepal district: secondary analysis of a population-based cross-sectional study
Source: Glob Ment Health (Camb). 2018 Nov 13;5:e37. doi: 10.1017/gmh.2018.28 (PMC6315279; doi:10.1017/gmh.2018.28)
Supplement: Supplementary file 1 [file S2054425118000286sup001.docx]

capture log close

log using "analysis.log", replace

*****************************

*** Nepal baseline CS AUD ***

*****************************

use C:\PRIME\CS\Nepal\CS_NE.dta, clear

svyset psu [pweight=pw], str(vdc)

********************

*** HOUSEKEEPING ***

********************

local myvars submissionid sid start idate received round ///

pw pwfull psu vdc fullint_ne ward ///

sex age rel caste_4c marit edu empcat_nepal econindex_3c ///

alcever aud? aud10 audpos tobac phqpos totalaud suithink whodas_simple hosp ///

audst__* auddisc* audtx audtx_* audtxcat* aud_4c

keep `myvars'

****************

*** NEW VARS ***

****************

recode age (min/29=0)(30/39=1)(40/49=2)(50/59=3)(60/max=4), gen(age_5c)

label define age5c 0 "18-29" 1 "30-39" 2 "40-49" 3 "50-59" 4 "60-88"

label values age_5c age5c

label variable age_5c "Age category (years)"

recode rel (1=1)(2=2)(3/77=77), gen(rel_3c)

label define rel3c 1 Hindu 2 Buddhist 77 Other

label values rel_3c rel3c

label variable rel_3c "Religion"

drop rel

recode marit (1=1)(2=2)(3/5=3), gen(marit_3c)

label define marit3c 1 Single 2 Married 3 "Post-marital"

label values marit_3c marit3c

label variable marit_3c "Marital status"

drop marit

recode edu (0/2=0)(3=1)(4=2), gen(edu_3c)

label define edu3c 0 "None-primary" 1 Secondary 2 "College/uni"

label values edu_3c edu3c

label variable edu_3c "Educational attainment"

drop edu

mvencode aud? aud1?, mv(.=0) override /* Imputes 0's due to skip pattern */

gen alcyear = 0

replace alcyear=1 if alcever==1 & totalaud>=1

label variable alcyear "Consumed alcohol, 12 months"

label values alcyear yesno

recode empcat_nepal (1=1)(2=2)(3/77=77), gen(emp_3c)

label define emp3c 1 Agriculture 2 "Service/business" 77 Other

label values emp_3c emp3c

label variable emp_3c "Occupation"

drop empcat_nepal

label define agree 0 "(Strongly) disagree" 1 "(Strongly) agree"

foreach var of varlist audst__* {

recode `var' (1/2=0)(3/4=1)

label values `var' agree

}

egen anystigma = anymatch(audst__*), v(1)

replace anystigma=. if !audpos

gen fwfull=int(pwfull)

* tab whodas_simple [fw=fwfull]

recode whodas_simple (min/12=1)(13/15=2)(16/max=3), gen(whodas_3c)

label variable whodas_3c "Functioning (WHODAS tertiles)"

label define whodas3c 1 High 2 Med 3 Low

label values whodas_3c whodas3c

egen cluster = group(vdc ward)

label variable cluster "Unique ward ID"

*bcskew0 bctotalaud = totalaud if sex==0 & alcyear==1

*label variable bctotalaud "Box Cox transf of totalaud"

*gen lntotalaud = ln(totalaud) if sex==0 & alcyear==1

*label variable lntotalaud "Log transf of totalaud"

**********************

*** START ANALYSIS ***

**********************

sum /* will need up upweight whodas econindex hosp for analysis */

svydes

table round, c(n idate min idate max idate)

tab sex, sum(alcever)

svy: prop alcever, over(sex)

***************

*** TABLE 1 ***

*************** Descriptives

* For Part 1 pts

tab sex

svy: tab sex

tab sex, sum(alcyear)

svy: prop alcyear, over(sex)

foreach i in 0 1 {

foreach var of varlist age_5c rel_3c caste_4c marit_3c edu_3 emp_3c tobac phqpos suithink {

tab sex if sex==`i'

tab `var' if sex==`i'

svy, subpop(if sex==`i'): tab `var'

svy, subpop(if sex==`i'): tab `var' alcyear, row pearson

}

}

* For Part 2 pts (make sure econindex is weighted before the category)

svyset psu [pweight=pwfull], str(vdc)

foreach i in 0 1 {

foreach var of varlist econindex_3c whodas_3c hosp {

tab sex if sex==`i'

tab `var' if sex==`i'

svy, subpop(if sex==`i'): tab `var'

svy, subpop(if sex==`i'): tab `var' alcyear , row

}

}

svyset psu [pweight=pw], str(vdc)

foreach i in 0 1 {

tab sex alcyear if sex==`i'

tab aud_4c if sex==`i' & alcyear==1

prop audpos if sex==`i' & alcyear==1

svy, subpop(if sex==`i' & alcyear==1): prop audpos

prop aud_4c if sex==`i' & alcyear==1

svy, subpop(if sex==`i' & alcyear==1): prop aud_4c

}

***************

*** TABLE 2 ***

*************** Mean AUDIT regressions

* Only the pw matters, not the strata.

* Therefore, can use the non-survey tnbreg as long as weights are included

* tab totalaud if sex==0 & alcyear

qui svy, subpop(if sex==0 & alcyear==1): tnbreg totalaud, ll(0)

margins `var', subpop(if sex==0 & alcyear==1) vce(unconditional)

*log using "meanaudit.csv", replace

*foreach var of varlist age_5c rel_3c caste marit_3c edu_3 emp_3c tobac phqpos suithink {

*svy, subpop(if sex==0 & alcyear==1): mean totalaud, over(`var')

*svy, subpop(if sex==0 & alcyear==1): regress totalaud i.`var'

*predict `var'hat,

*gen `var'resid = totalaud - `var'hat

* svy, subpop(if sex==0 & alcyear==1): tnbreg totalaud i.`var', ll(0)

*predict double `var'hat if sex==0 & alcyear==1, cm

*gen `var'resid = totalaud - `var'hat if sex==0 & alcyear==1

*graph box `var'resid if sex==0 & alcyear==1 [pw=pw], over(`varhat')

* qui margins `var', subpop(if sex==0 & alcyear==1) vce(unconditional) contrast

*qui levelsof `var'

*foreach value in `r(levels)' {

* lincom _cons + `value'.`var', cformat(%4.3g) irr

* disp "`var'" "=" "`: label (`var') `value''" "," r(estimate) "," r(se)

* }

*}

*log close

capture program drop myboot

program myboot, rclass

tnbreg totalaud `1'.`2' if sex==0 & alcyear==1 [pw=`3'], ll(0)

lincom _cons+`1'.`2', irr

matrix b=r(estimate)

local b=el(b,1,1)

return scalar beta=`b'

end

foreach var of varlist age_5c rel_3c caste marit_3c edu_3 emp_3c tobac phqpos suithink {

qui levelsof `var'

foreach value in `r(levels)' {

qui bootstrap b=r(beta), reps(1000) : myboot `value' `var' pw /* uses pw for full sample */

estat bootstrap, p

}

}

foreach var of varlist econindex_3c hosp whodas_3c {

qui levelsof `var'

foreach value in `r(levels)' {

qui bootstrap b=r(beta), reps(1000) : myboot `value' `var' pwfull /* uses pwfull for subsample */

estat bootstrap, p

}

}

***************

*** TABLE 3 ***

*************** Contact coverage, discussion and stigma

svyset psu [pweight=pw], str(vdc)

tab audtx round if sex==0 & audpos, col

prop audtx if sex==0 & audpos, over(round)

svy, subpop(if sex==0 & audpos): proportion audtx, over(round)

svy, subpop(if sex==0 & audpos): tab round audtx, row

mrtab audtx_* if sex==0 & audtx, incl sort desc by(round)

mrtab audtxcat_* if sex==0 & audpos==1, incl sort desc by(round)

svy, subpop(if sex==0 & audpos==1): tab round audtx, row

svy, subpop(if sex==0 & audpos==1): tab round auddisc, row

foreach var of varlist auddisc_* {

svy, subpop(if sex==0 & audpos): tab `var'

}

tab anystigma if sex==0

mrtab audst__* if sex==0 [aw=pw], incl sort desc f(%9.3g)

svy, subpop(if sex==0): tab anystigma

foreach var of varlist audst__* {

svy, subpop(if sex==0): tab `var'

}

**************

*** GRAPHS ***

**************

/*import delimited "meanaudit.csv", delimiter(comma) rowrange(19:45) clear

encode v1, gen(varlabel)

rename v2 coef

rename v3 se

gen irr=exp(coef)

gen lb=exp(coef-invnormal(0.975)*se)

gen ub=exp(coef+invnormal(0.975)*se)

drop if v1=="rel_3c=Other"

drop if v1=="marit_3c=Post-marital"

graph twoway (scatter irr varlabel, ytitle("Mean AUDIT score") yline(5.500797) mcolor(black) legend(off)) ///

(rcap lb ub varlabel, lcolor(black) xlabel(#29, labsize(small) valuelabel angle(45)))

*/
